# Cationic Gemini Surfactants in the Oil Industry: Applications in Extraction, Transportation and Refinery Products

**DOI:** 10.3390/molecules31010108

**Published:** 2025-12-27

**Authors:** Bogumił Brycki, Adrianna Szulc, Justyna Brycka, Iwona Kowalczyk

**Affiliations:** 1 Department of Bioactive Products, Faculty of Chemistry, Adam Mickiewicz University Poznan, Uniwersytetu Poznańskiego 8, 61-614 Poznan, Poland; iwkow@amu.edu.pl; 2 MDA Sp. z o.o., Wolczynska 18, 60-003 Poznan, Poland; adrszu@gmail.com (A.S.); biuro@mda.org.pl (J.B.)

**Keywords:** gemini surfactants, oil industry, enhanced oil recovery, corrosion inhibitors

## Abstract

The petroleum industry faces intensifying challenges related to the depletion of easily accessible reservoirs and the growing energy demand, necessitating the adoption of advanced chemical agents that can operate under extreme conditions. Cationic gemini surfactants, characterized by their unique dimeric architecture consisting of two hydrophilic head groups and two hydrophobic tails, have emerged as superior alternatives to conventional monomeric surfactants due to their enhanced interfacial activity and physicochemical resilience. This review provides a comprehensive analysis of the literature concerning the molecular structure, synthesis, and functional applications of cationic gemini surfactants across the entire oil value chain, from extraction to refining. The analysis reveals that gemini surfactants exhibit critical micelle concentrations significantly lower than their monomeric analogs and maintain stability in high-temperature and high-salinity environments. They demonstrate exceptional efficacy in enhanced oil recovery through ultra-low interfacial tension reduction and wettability alteration, while simultaneously serving as effective drag reducers, wax inhibitors, and dual-action biocidal corrosion inhibitors in transportation pipelines. Cationic gemini surfactants represent a transformative class of multifunctional materials for the oil industry.

## 1. Introduction

Crude oil remains one of the most strategically significant resources in the global economy, providing the foundation for energy generation, chemical manufacturing, and transportation fuels. Despite increasing investment in renewable energy sources, petroleum still accounts for approximately one-third of global primary energy consumption and remains indispensable for the production of lubricants, polymers, and specialty chemicals. The growing energy demand, combined with the progressive depletion of easily accessible reservoirs, has intensified the need for more efficient, sustainable, and economically viable methods of crude oil extraction, transportation, and refining. As conventional production declines, enhancing oil recovery (EOR), reducing transportation losses, and improving refinery efficiency have become critical technological and financial priorities [1,2,3,4].

**Figure 1:**
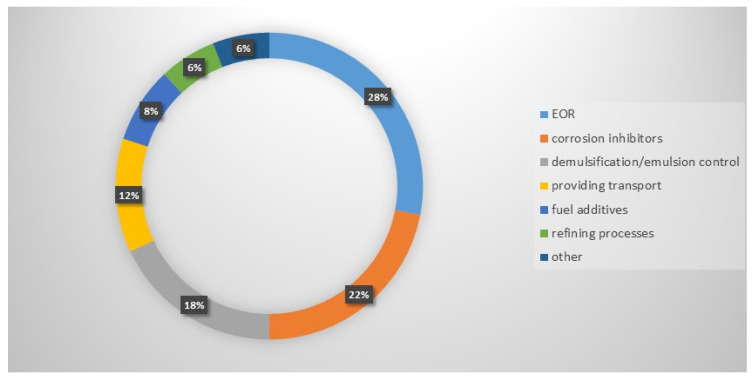
Distribution of Gemini surfactant applications in the petroleum industry.

Surfactants play a pivotal role in nearly every stage of the petroleum value chain due to their amphiphilic nature, which allows them to reduce interfacial tension, alter wettability, stabilize emulsions, and facilitate phase separation. In extraction processes, surfactant flooding can mobilize residual oil trapped within porous rock formations, significantly improving displacement efficiency beyond that achievable by waterflooding alone. Industrial data indicate that the addition of surfactant formulations may increase oil recovery by 5–20% of the original oil in place, depending on reservoir conditions. From an economic perspective, the incremental cost of surfactant-based recovery typically ranges from USD 1 to USD 5 per barrel of additional oil, while the potential production gain often exceeds 10–15 barrels per dollar invested, representing a substantial return on investment. Furthermore, during crude transportation, surfactants can reduce drag, improve the flow of heavy and viscous crudes, and minimize pipeline energy consumption—yielding operational cost savings that may reach 10–25% in pumping energy. In refining, surfactants assist in demulsification, corrosion inhibition, and catalytic phase transfer, leading to improved separation efficiency and lower chemical usage [3,4,5,6]. The distribution of Gemini surfactant applications in the petroleum industry highlights their versatility (Figure 1). Enhanced Oil Recovery (EOR) represents the largest share at 28%, followed by corrosion inhibition (22%) and demulsification/emulsion control (18%). Other notable uses include facilitating transport (12%), fuel additives (8%), and refining processes (6%), with a small fraction allocated to miscellaneous applications (6%). This distribution underscores the multifunctional nature of Gemini surfactants across different operational needs in the oil industry.

Among the diverse classes of surface-active agents, cationic gemini surfactants have emerged as a particularly promising subclass due to their unique molecular topology and superior interfacial behavior. Cationic gemini surfactants (dimeric quaternary ammonium salts) can be varied to tailor physicochemical properties such as micelle size, aggregation number, and interfacial packing. This architecture results in critical micelle concentrations (CMCs) one to two orders of magnitude lower than those of their monomeric analogs, accompanied by markedly enhanced surface activity and thermal stability [7,8,9,10]. Cationic gemini surfactants exhibit exceptional tolerance to high salinity, pressure, and temperature—conditions frequently encountered in petroleum reservoirs. Their dual-head structure also facilitates stronger adsorption onto negatively charged mineral surfaces, promoting effective wettability alteration from oil-wet to water-wet states, which is critical for enhanced recovery. Recent studies have demonstrated that gemini surfactants can maintain efficient interfacial tension reduction (<10^−3^ mN/m) even under high brine concentrations, outperforming conventional quaternary ammonium surfactants. Additionally, the modular nature of their synthesis enables molecular design optimization through variation in spacer length, counterion type, and alkyl chain composition, allowing for targeted tuning toward specific oilfield applications [11,12]. Multifunctional gemini surfactants, apart from their superior surface-active properties, exhibit pronounced antimicrobial and anticorrosive activities. Their amphiphilic dimeric structure facilitates strong interactions with microbial cell membranes, leading to effective disruption and inhibition of microbial growth [13,14,15,16,17,18]. Simultaneously, their ability to adsorb onto metal surfaces forms protective layers that significantly reduce corrosion processes [19,20,21,22,23]. As a result of these combined effects, gemini surfactants demonstrate a remarkable capability to inhibit biocorrosion, which is of particular importance in the oil industry where microbial-induced degradation of materials poses serious operational challenges [24].

Despite their promising performance characteristics, large-scale industrial implementation of cationic gemini surfactants remains constrained by several factors, including relatively high synthesis costs, limited commercial availability, and environmental concerns related to biodegradability and toxicity [25,26]. Current research therefore focuses on developing green gemini surfactants derived from renewable feedstocks and incorporating biodegradable spacers or counterions, aiming to balance performance with environmental compatibility [27,28]. Advances in computational modeling, molecular dynamics simulations, and experimental formulation optimization are expected to accelerate the design of next-generation gemini surfactants tailored for oilfield applications [29,30,31].

Consequently, this review aims to provide a comprehensive analysis of cationic gemini surfactants within the oil industry, encompassing their molecular structure, physicochemical behavior, and diverse functional roles across extraction, transportation, and refinery processes. Special emphasis is placed on mechanistic understanding, and the elucidation of their dual capabilities in interface modification and asset protection. Furthermore, the discussion addresses the economic implications and sustainability perspectives of these compounds, evaluating their potential as key enabling materials for more efficient and environmentally responsible petroleum production.

## 2. Discussion

### 2.1. Structure and Physicochemical Characteristics of Cationic Gemini Surfactants

#### 2.1.1. Molecular Architecture

**Figure 2:**
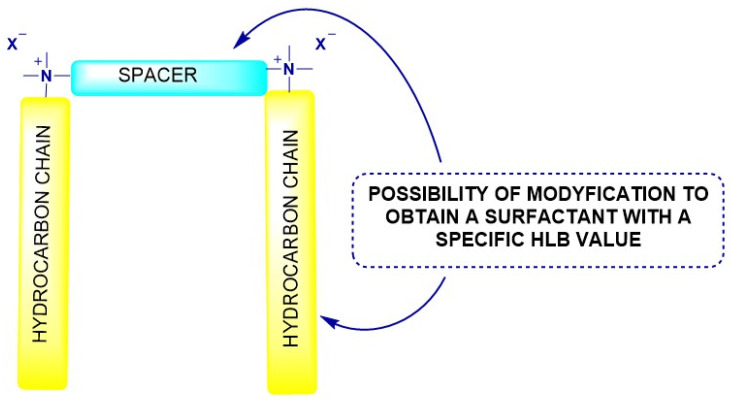
Structural diagram of gemini surfactants.

Cationic gemini surfactants are a distinct class of amphiphilic compounds characterized by two hydrophobic alkyl tails and two cationic head groups linked by a covalent spacer (Figure 2) [7]. This unique configuration gives rise to cooperative interactions between the two polar heads and hydrophobic chains, resulting in physicochemical behaviors that differ markedly from conventional single-chain surfactants. The presence of two ionic centers in close proximity significantly enhances electrostatic interactions and reduces molecular mobility at interfaces, leading to the formation of dense, well-ordered surface films. The spacer plays a pivotal role in defining the physicochemical and interfacial behavior of gemini surfactants. Its length, rigidity, and chemical composition determine the optimal molecular geometry, packing, and micelle formation tendency. Short aliphatic spacers promote compact molecular conformations, favoring strong electrostatic interactions between the head groups and reduced molecular flexibility. Conversely, longer or more flexible spacers (e.g., polyether, amide, or aromatic types) enhance the spatial separation between the cationic centers, reducing electrostatic repulsion and facilitating better solubility and aggregation in aqueous systems. In addition to the spacer, the length of the hydrophobic substituents (alkyl chains) exerts a pronounced influence on the hydrophilic–lipophilic balance (HLB) and overall surfactant performance. Increasing the chain length enhances hydrophobic interactions, reduces the CMC, and favors adsorption at nonpolar interfaces, but may also decrease solubility in aqueous media. Shorter chains, in contrast, promote higher water solubility and easier micelle formation but may weaken interfacial film stability [7,32]. Therefore, careful optimization of alkyl chain length—typically ranging from C_8_ to C_18_—allows fine-tuning of amphiphilicity for specific petroleum applications.

**Figure 3:**
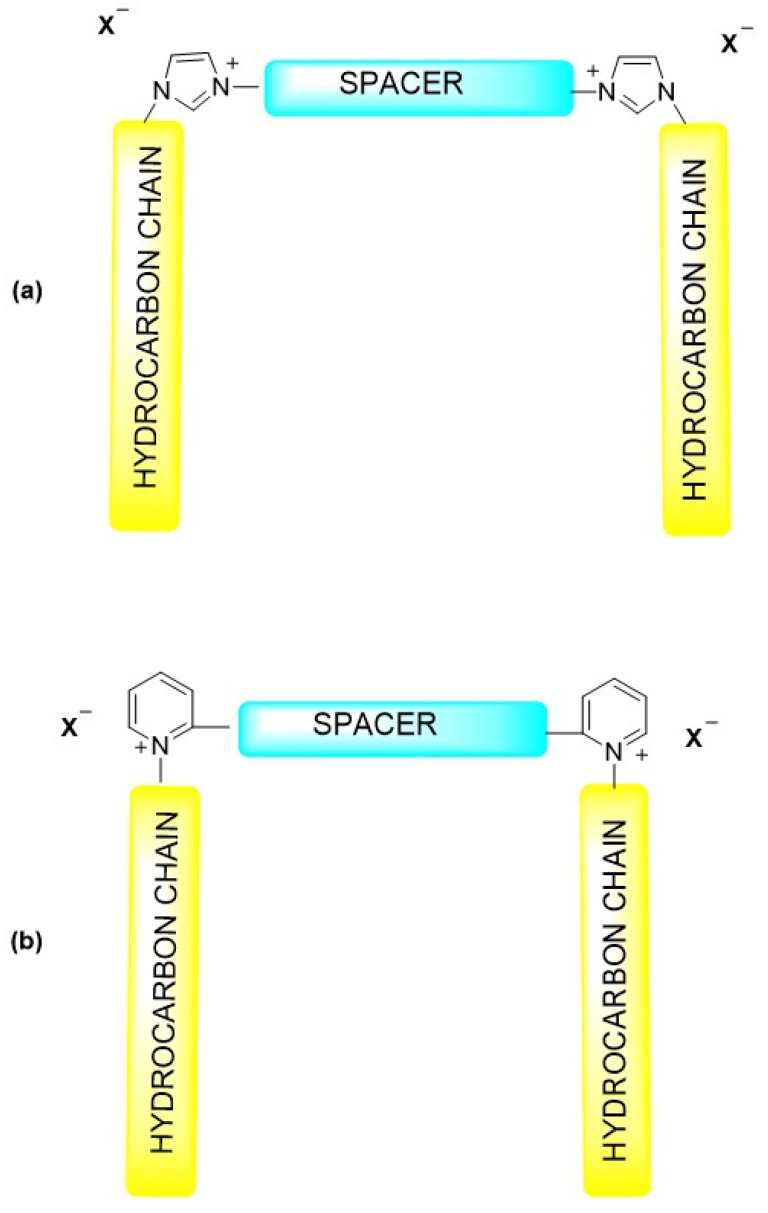
Structural diagram of (**a**) imidazolium- and (**b**) pyridinium-based gemini surfactants.

Moreover, both the spacer and the hydrophobic substituents can be chemically modified with a wide variety of organic groups, including ether, ester, amide, aromatic, or heterocyclic moieties. Such modifications allow the introduction of functional groups that can respond to changes in pH, temperature, or ionic strength, leading to tunable interfacial behavior. For example, introducing ether or amide linkages increases hydrophilicity and flexibility, while aromatic or fluorinated substituents enhance rigidity and interfacial stability in nonpolar systems. These structural variations not only alter HLB values but also influence adsorption dynamics, aggregation morphology, and compatibility with mineral surfaces and crude oil components [7,33]. Figure 3 presents the molecular structures of imidazolium- and pyridinium-based gemini surfactants, which are among the most commonly used systems in the oil industry [34]. The presence of the spacer and hydrocarbon tails illustrates the broad possibilities for structural modification, analogous to those observed for conventional gemini surfactants. Such variability enables fine-tuning of physicochemical properties to meet specific application requirements.

The chemical nature of both the spacer and substituents thus determines the balance between hydrophilicity and hydrophobicity, directly affecting solubility, micellization behavior, and interfacial efficiency. This structural tunability provides a powerful molecular design tool, enabling the synthesis of gemini surfactants specifically tailored for complex oilfield environments [30,35].

#### 2.1.2. Comparison with Conventional (Monomeric) Surfactants

In contrast to monomeric surfactants, gemini molecules display significantly improved surface and interfacial activity, arising from cooperative intramolecular and intermolecular effects. Experimental studies consistently demonstrate that the CMC of gemini surfactants is typically 10–100 times lower than that of their single-chain analogs. This reduction reflects a more favorable balance of hydrophobic and electrostatic interactions, as the dual-chain structure promotes micellization at lower concentrations [36,37,38,39]. The improved efficiency of gemini surfactants is also evident in their ability to achieve lower surface and interfacial tensions. At minimal dosages, these surfactants can reduce surface tension values to approximately 28–32 mN·m^−1^ and interfacial tension between oil and water to below 10^−2^ mN·m^−1^. Such performance translates directly into enhanced emulsification, wettability modification, and interfacial stabilization—all critical in petroleum extraction and refining [12]. Structurally, the dual-head arrangement provides stronger adsorption onto negatively charged substrates such as silicate or carbonate rock surfaces, resulting in greater surface coverage and durability. Furthermore, gemini surfactants exhibit remarkable stability under harsh reservoir conditions, including elevated temperature, high pressure, and high salinity. Their ability to maintain structural integrity and functional activity in the presence of multivalent ions (e.g., Ca^2+^, Mg^2+^) or high ionic strength brines distinguishes them from most conventional cationic agents, whose performance often declines sharply under similar conditions [40]. Another key advantage lies in their multifunctionality: while conventional surfactants serve primarily as emulsifiers or dispersants, gemini surfactants can act simultaneously as corrosion inhibitors, scale suppressors, or flow improvers due to their strong adsorption and persistent interfacial films. This combination of high efficiency and multifunctional behavior underpins the growing industrial interest in these compounds [7].

#### 2.1.3. Surface Activity and Critical Micelle Concentration (CMC)

The surface activity of gemini surfactants is primarily governed by their molecular symmetry and cooperative interactions between the twin hydrophobic tails and dual cationic heads. When dissolved in aqueous or mixed media, these molecules rapidly migrate to interfaces, where they orient themselves such that the hydrophobic tails extend into the nonpolar phase and the cationic heads remain solvated in the aqueous phase. This orientation results in the formation of compact, highly ordered interfacial layers that efficiently reduce surface free energy. The low CMC (Table 1) values characteristic of gemini surfactants stem from strong hydrophobic interactions and minimized electrostatic repulsion between adjacent head groups, facilitated by the presence of the spacer. This results in earlier onset of micellization and the formation of micelles with smaller aggregation numbers compared to monomeric analogs. For many gemini systems, CMC values range between 10^−3^ and 10^−6^ mol·L^−1^, demonstrating their exceptional efficiency [41,42,43,44,45].

Thermodynamically, micellization of gemini surfactants is driven by a large negative Gibbs free energy (ΔG°_mic_), indicating spontaneous self-assembly. Parameters such as the maximum surface excess (Γ_max_) and minimum molecular area highlight their dense interfacial packing, often approaching theoretical close-packing limits. The presence of two hydrophobic chains allows tighter micellar cores and more stable aggregates, contributing to superior emulsification and phase behavior in oil–water systems. These features make gemini surfactants ideal candidates for applications requiring strong interfacial stabilization and efficient phase modification at minimal concentrations [7,42,46].

#### 2.1.4. Micellar and Aggregation Behavior in Hydrocarbon Media

The aggregation behavior of cationic gemini surfactants in hydrocarbon-rich or mixed aqueous–hydrocarbon media is complex and depends strongly on molecular architecture, spacer design, solvent polarity, and temperature. In aqueous solutions, gemini surfactants initially form spherical micelles, which may evolve into cylindrical, wormlike, or vesicular structures as concentration increases. The dual hydrophobic tails promote closer packing within micellar cores, yielding smaller, more stable aggregates than those formed by monomeric surfactants. In nonpolar or hydrocarbon-dominant environments, gemini surfactants can form reverse micelles, where the hydrophilic head groups cluster inward, solvating small water droplets or polar impurities within the micellar core. These reverse structures play a vital role in stabilizing water-in-oil emulsions and solubilizing trace polar compounds during crude oil processing. Furthermore, the combination of cationic charge and amphiphilic balance enables gemini surfactants to form hierarchical assemblies such as vesicles, bilayers, and liquid crystalline phases under specific conditions of temperature and salinity. The spacer length and flexibility are critical in determining aggregation morphology. Short spacers favor compact micelles with limited hydration, while longer spacers increase inter-head distance, allowing for greater curvature and more complex structures. This tunable aggregation behavior provides valuable control over emulsion properties, viscosity, and drag reduction—all essential parameters in extraction and transportation processes within the oil industry [7,47,48,49,50,51].

### 2.2. Mechanistic Aspects of Interaction with Oil and Rock Surfaces

#### 2.2.1. Adsorption Mechanisms on Mineral and Reservoir Rock Surfaces

The adsorption of cationic gemini surfactants onto mineral and reservoir rock surfaces represents a fundamental process governing their performance in oilfield applications. The interaction is mainly controlled by electrostatic attraction between positively charged quaternary ammonium or imidazolium head groups and negatively charged sites on mineral surfaces such as silicates, aluminosilicates, or carbonates. However, the overall adsorption process is multifactorial—it involves not only Coulombic forces but also van der Waals interactions, hydrogen bonding, and hydrophobic association. The presence of two cationic head groups enhances surface binding affinity and promotes the formation of multilayer or bilayer structures, depending on surfactant concentration and ionic strength. The molecular geometry, especially the nature of the spacer, determines the configuration of adsorbed layers. Short spacers favor compact adsorption and high surface coverage, whereas longer, more flexible spacers allow for adaptive conformations, optimizing contact with surface heterogeneities. The result is the formation of dense, stable interfacial films that remain intact under shear flow, salinity, and temperature fluctuations. Compared to conventional monomeric surfactants, gemini species exhibit slower desorption kinetics and higher resistance to displacement, providing long-lasting surface modification effects [52,53,54].

#### 2.2.2. Compatibility with Crude Oil Components and Brine

Cationic gemini surfactants operate within complex chemical environments where they interact not only with mineral surfaces but also with crude oil constituents and brine ions. Their performance is particularly influenced by interactions with asphaltenes, resins, and paraffinic compounds. Due to their positive charge, gemini surfactants can electrostatically bind to acidic asphaltenes, disrupting aggregation and deposition processes that often lead to pore blockage and pipeline fouling. This dispersion effect is critical for maintaining flow assurance and preventing formation damage. At the same time, the molecular design of gemini surfactants allows controlled emulsification or demulsification, depending on operational requirements. In production processes, water-in-oil emulsions can be stabilized to enhance oil mobility, whereas in refinery dehydration stages, gemini surfactants can be tailored to promote rapid phase separation. Brine composition exerts an additional layer of influence. Divalent cations such as Ca^2+^ and Mg^2+^ can compress the electrical double layer, promoting stronger adsorption and improved film compactness. Conversely, excessive salinity can screen electrostatic interactions, reducing adsorption efficiency. Nonetheless, the intrinsic structural robustness of gemini surfactants allows them to maintain function under brine salinities exceeding those tolerated by conventional surfactants. This adaptability under high ionic strength conditions is a decisive advantage in offshore and high-salinity reservoir applications [55,56,57,58].

#### 2.2.3. Stability Under Reservoir Conditions

Reservoir environments impose extreme physical and chemical challenges—high temperatures (up to 120–150 °C), pressures exceeding 20 MPa, and brine salinities approaching saturation. Under such conditions, surfactant degradation or desorption often limits long-term performance. Gemini surfactants, however, demonstrate remarkable resilience due to their molecular symmetry, strong headgroup binding, and cooperative hydrophobic interactions [59].

Thermal stability is enhanced by the reduced CMC and tight packing of dual-chain aggregates, which limit molecular motion and prevent breakdown. The spacer unit contributes to flexibility and resilience, allowing the surfactant to accommodate structural stress without dissociation. High-pressure flow experiments have shown that gemini surfactants form quasi-permanent adsorption layers that resist desorption even under continuous water flooding. These interfacial films not only preserve wettability alteration and IFT reduction effects but also serve as protective barriers against corrosion and scale formation on metallic and mineral surfaces. Moreover, the kinetic stability of gemini surfactant aggregates enables consistent performance during long-term injection cycles. Their persistence under harsh conditions makes them ideal candidates for tertiary recovery methods and long-duration treatments in reservoirs where conventional surfactants fail [1,60,61,62].

### 2.3. Applications in the Oil Extraction Process

#### 2.3.1. Enhanced Oil Recovery Techniques Using Gemini Surfactants

Compared with conventional monomeric surfactants, gemini surfactants demonstrate markedly superior performance in EOR, primarily due to their distinctive dimeric architecture and the resulting physicochemical properties. The exceptionally low CMC allows gemini surfactants to achieve ultra-low oil–water interfacial tension under salinity and temperature conditions in which conventional surfactants become ineffective or require impractically high dosages. Gemini surfactants also exhibit enhanced tolerance to high salinity and the presence of divalent cations, which commonly destabilize or precipitate monomeric surfactants. Their dual headgroups and two hydrophobic chains promote robust interfacial adsorption and prevent salt-induced collapse of micelles, enabling stable performance in high-salinity and high-temperature (HT–HS) reservoirs typical of carbonate and sandstone formations at advanced depletion stages. This physicochemical robustness is further reinforced by the ability of gemini surfactants to form complex self-assembled structures, including elongated worm-like micelles, which can impart viscoelasticity to the injected fluid. Such behavior reduces viscous fingering, improves mobility control, and enhances the sweep efficiency of the displacement front—effects that monomeric surfactants typically fail to achieve without polymeric co-additives [30].

Based on the extracted content, gemini surfactants exhibit the highest overall potential for EOR because they combine extremely low CMC with strong thermal and salinity tolerance, as well as the ability to form complex self-assembled structures that enhance sweep efficiency and interfacial performance (Table 2). Zwitterionic and nonionic surfactants offer complementary advantages—particularly in terms of stability and environmental compatibility—and can synergistically enhance gemini-based formulations. Conventional monomeric surfactants, despite their historical use, demonstrate lower overall performance and exhibit significant limitations under challenging reservoir conditions [60].

Al-Azani et al. described the application of a gemini surfactant for enhanced oil recovery under harsh carbonate reservoir conditions. Their results demonstrate a clear superiority of the gemini surfactant relative to conventional anionic and cationic surfactants typically employed in carbonates. Notably, the gemini system achieved substantial wettability alteration, decreasing the contact angle from strongly oil-wet values exceeding 130° to below 90°, with a maximum shift of approximately 81° at 1500–2500 ppm. In contrast, conventional cationic surfactants such as DTAB generally require higher concentrations and still generate more limited wettability change, while anionic sulfonates—despite sometimes outperforming cationics—often suffer from excessive adsorption. Coreflooding results further highlight the performance benefits of Gemini surfactants: after seawater flooding recovered 55% of the original oil in place, tertiary injection of a 2500 ppm Gemini surfactant yielded an additional ~17% OOIP, raising ultimate recovery to ~72%. This incremental gain is substantially higher than values typically reported for single-component surfactant floods in high-temperature (>100 °C), high-salinity (>200,000 ppm TDS) carbonate systems. Moreover, the dynamic retention of only 0.51 mg/g-rock remains below the 0.7 mg/g-rock values often used in field-pilot design, underscoring the practicality of gemini-based formulations [63].

**Figure 4:**
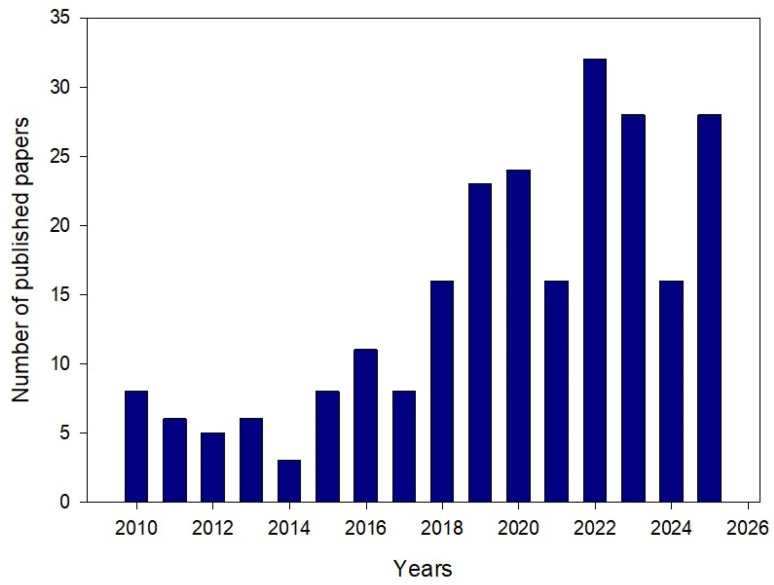
Number of published papers on the use of gemini surfactants in EOR versus the year of publication (based on Scopus).

Liu and co-workers demonstrated a strong synergistic effect between the gemini surfactant and the anionic surfactant (AES; sodium dodecyl polyoxyethylene ether sulfate), showing that mixed systems outperform individual components in all key EOR metrics. The optimal mixture reached an interaction parameter stronger than typical cationic–anionic pairs—and produced ultralow IFT values of 10^−3^ mN/m at only 200 mg/L, compared with 0.091 mN/m (gemini) and 0.89 mN/m (AES) alone. The blend also enhanced wettability alteration (contact angle reduced to 33.6° vs. 64–72° individually) and nearly doubled the incremental oil recovery, achieving 20.37% compared with the lower values obtained from single-surfactant flooding. These results confirm that gemini-based hybrid formulations provide superior performance in chemical EOR [64]. Great interest has been reflected by the increasing number of publications on the use of gemini surfactants in EOR (Figure 4).

Gemini surfactants exhibit significantly better numerical performance than other surfactant classes across all major criteria relevant to EOR (Table 3). Their CMC values are typically one to two orders of magnitude lower than those of monomeric surfactants, enabling ultra-low interfacial tension levels (<10^−3^ mN/m) at minimal dosage. They also maintain strong interfacial activity under high-temperature and high-salinity conditions (up to 150 °C), conditions in which many conventional surfactants fail due to precipitation or micellar collapse. These attributes translate into superior incremental oil recovery, often exceeding 30% OOIP (original oil in place) in controlled laboratory flooding experiments, thus placing gemini surfactants among the most powerful chemical EOR agents currently investigated [65,66,67,68,69].

#### 2.3.2. Foam and Emulsion Stabilization in Drilling Fluids

Gemini surfactants have emerged as particularly effective stabilizers of foams and emulsions in drilling fluids because their dimeric architecture promotes dense interfacial packing, stronger intermolecular cohesion and the formation of viscoelastic interfacial films that retard drainage and coalescence [70]. For example, an eco-friendly gemini surfactant with ester and ether groups in substituents increased foam half-life by ~78% and raised initial foam volume by ~21% compared with a commercial foaming blend under laboratory conditions, demonstrating both greater foam longevity and generation efficiency relevant to underbalanced drilling [71]. In mixed or nanoparticle-augmented systems, gemini surfactants further enhance stability and salt/temperature tolerance: catanionic or gemini–anionic mixtures have been shown to sustain ultralow IFT and stable foams at salinities typical of produced waters, and gemini-stabilized foams resist coarsening in the presence of divalent ions and elevated temperatures encountered downhole [72]. For emulsion drilling fluids, polyhydroxy and other functionalized gemini surfactants improve inverted-emulsion rheology and mechano-responsive behavior, producing emulsions with higher shear-recovery and delayed phase separation relative to conventional single-chain emulsifiers—a desirable trait for cuttings transport and pumpability [70]. Collectively, these studies indicate that gemini surfactants markedly increase foam half-life and initial foamability, enable stable emulsions under high salinity/temperature, and offer tunable molecular handles (spacer length, headgroup chemistry) to optimize drilling-fluid performance for specific well conditions.

### 2.4. Applications in Transportation and Pipeline Operations

#### 2.4.1. Flow Assurance and Drag Reduction

Gemini surfactants have recently gained attention as promising drag-reducing agents (DRAs) during crude oil transportation through pipelines. The unique double-tail/double-head architecture facilitates the formation of worm-like micelles or viscoelastic micellar networks under flow conditions, which can damp turbulence, reduce frictional pressure loss, and decrease the apparent viscosity of heavy crude oils. Indeed, studies reviewed in the most recent comprehensive survey report substantial reductions in turbulence and energy losses when gemini surfactants are added to transport media, thereby improving flow assurance and lowering pumping costs [72,73]. In particular, in heavy oil systems with high viscosity and under low-temperature conditions, the addition of gemini surfactants (sometimes in combination with nanoparticles) has led to reported crude-oil viscosity reductions up to 85–92% at 10 °C—a level of performance that significantly enhances pipeline operability and mitigates issues related to deposition and pipe blockage [58,74].

In the context of pipeline transportation, gemini surfactants can also function as effective pour point depressants (PPDs) and wax inhibitors. Their long alkyl chains (typically C16–C18) facilitate cocrystallization with paraffin waxes, altering crystal morphology from large, interlocking structures to smaller, spherical particles. This modification reduces gel strength, lowers the pour point, and minimizes wax deposition on pipeline walls. Additionally, the aromatic or heteroaromatic components in some gemini structures can interact with asphaltenes via π–π stacking and hydrogen bonding, helping to disperse asphaltene aggregates that otherwise exacerbate wax deposition. Studies indicate that gemini surfactants with medium spacer groups (e.g., pentamethylene) often demonstrate better performance due to improved molecular flexibility and packing at the oil–water interface. Their efficiency in reducing the consumption of cosurfactants (e.g., 1-butanol) in microemulsion formulations further highlights their potential for creating stable, low-viscosity fuel blends that are pumpable at lower temperatures [55,75].

Gemini surfactants, characterized by their unique molecular structure offer enhanced performance in wax deposition control during crude oil transportation. Their superior surface activity and ability to significantly reduce interfacial tension make them effective wax dispersants and crystal growth inhibitors. In pipeline operations, they are utilized to modify wax crystal morphology, reduce oil viscosity, and improve low-temperature flowability. When combined with solvents or polymeric inhibitors, Gemini surfactants further enhance wax inhibition, supporting flow assurance and reducing the frequency of mechanical interventions such as pigging. Their efficiency in stabilizing waxy crude oils under cold conditions highlights their potential as advanced chemical additives for sustainable and cost-effective pipeline maintenance [76].

#### 2.4.2. Corrosion Inhibition in Pipeline Systems

**Figure 5:**
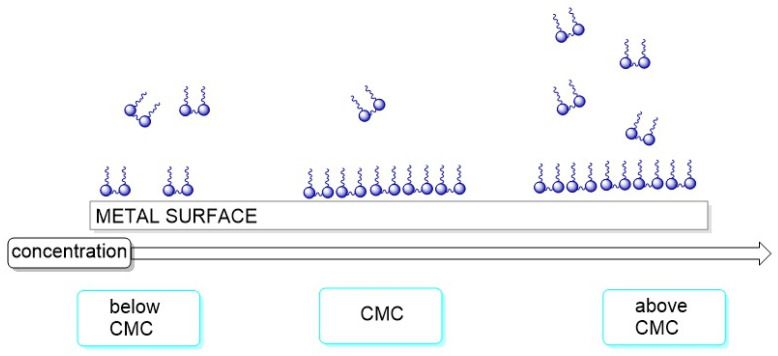
Anticorrosion mechanism of gemini surfactants depending on the concentration. 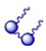 is gemini surfactants. The arrow indicates increasing concentration of gemini surfactant.

Corrosion of carbon steel pipelines and infrastructure remains a persistent problem in oil and gas transport, particularly in environments with acidic media, high salinity, or CO_2_/H_2_S presence. The deterioration of metal pipelines due to general corrosion is primarily an electrochemical process, where the metal, acting as an anode, is oxidized, and a cathodic reaction, typically the reduction of oxygen or hydrogen ions, occurs simultaneously [77]. This process severely compromises the structural integrity and operational lifespan of the pipeline infrastructure. Gemini surfactants have shown significant potential as corrosion inhibitors in such contexts [19]. The primary anti-corrosion mechanism of gemini surfactants involves forming a compact, dense protective layer on the metal surface (Figure 5). This layer acts as a physical barrier, effectively blocking the access of aggressive species (such as chloride or hydrogen ions) to the metal, thus significantly decreasing the corrosion rate. The adsorption can occur via physisorption (involving weak van der Waals forces or electrostatic attraction) or chemisorption (involving the sharing or transfer of electrons between the gemini molecule and the metal surface), depending on the specific surfactant structure and the corrosive medium [19,78,79].

Studies frequently demonstrate the high efficacy of GSs. For example, a quaternary ammonium GS with a long alkyl chain has been reported to achieve a corrosion inhibition efficiency (CIE) exceeding 95% for carbon steel in a 1 M HCl solution at a concentration as low as 50 ppm at 298 K. This is often verified using electrochemical techniques such as Tafel polarization curves and electrochemical impedance spectroscopy (EIS), which show a decrease in corrosion current density (Icorr) and an increase in charge transfer resistance (Rct) upon the addition of the GS inhibitor [80,81].

A significant threat to pipeline systems is microbiologically influenced corrosion (MIC), often termed biocorrosion. MIC is initiated and accelerated by the metabolic activities of microorganisms, particularly sulfate-reducing bacteria (SRB), which form dense communities known as biofilms on the pipeline surface. Biofilms create localized anaerobic environments, leading to aggressive pitting corrosion. The use of gemini surfactants is particularly advantageous because they offer a crucial dual-action capability: corrosion inhibition and antimicrobial activity. The same dicationic structure that enables effective adsorption onto the metal also provides potent biocidal properties. The positively charged head groups of the gemini surfactants strongly interact with the negatively charged bacterial cell membrane, leading to membrane disruption, leakage of cellular contents, and ultimately, cell death [7]. By eradicating the biofilm-forming bacteria and preventing the initial attachment of microbes, gemini surfactants effectively mitigate biocorrosion. This makes them superior to traditional single-function inhibitors, as they address both the electrochemical and biological components of pipeline degradation, offering a holistic approach to pipeline integrity management [82,83].

### 2.5. Additives in Fuel Formulations and Lubricants

Gemini surfactants have attracted considerable interest as multifunctional additives in both fuel formulations and lubricant systems due to their exceptional surface activity, molecular tunability, and strong tendency to form stable interfacial films. Their dimeric configuration facilitates more effective adsorption onto hydrocarbon–water or hydrocarbon–metal interfaces compared with conventional single-chain surfactants, enabling enhanced detergency, dispersancy, and stabilization of multiphase fuel systems. In diesel fuels, gemini surfactants function as efficient wax dispersants and cold-flow improvers, reducing wax crystal size and preventing agglomeration at low temperatures. Maithufi et al. demonstrated that incorporating gemini surfactants into diesel significantly decreased the cold-filter plugging point (CFPP), indicating improved low-temperature operability and reduced risk of pipeline blockage during winter conditions [84]. These compounds also enhance the stability of fuel microemulsions and improve the solubilization of polar trace contaminants, contributing to cleaner combustion and reduced deposit formation in injection systems.

Gemini surfactants, particularly those derived from ionic liquids (ILGS), represent a promising class of additives for advanced fuel formulations and lubricants, due to their unique molecular architecture and enhanced interfacial activity. In fuel applications, their incorporation into diesel/vegetable oil blend microemulsions significantly improves thermodynamic stability, reduces the required amount of co-surfactant, and enables the emulsification of water, which can lead to more efficient combustion and lower emissions of NOx, particulate matter, and unburned hydrocarbons [85]. The tunable spacer length allows for optimization of micelle formation and emulsion stability, with longer spacers (e.g., pentamethylene) often yielding better performance, smaller nano-droplet sizes, and favorable viscosity profiles comparable to conventional diesel) [86,87]. In lubricant formulations, the strong adsorption and film-forming capabilities of Gemini surfactants can enhance extreme pressure performance, reduce friction, and improve dispersion of contaminants, although specific studies in this area remain an emerging field. Their dual functionality and structural adaptability make ILGS-based Gemini surfactants a versatile additive platform for developing next-generation, environmentally benign fuels and high-performance lubricants [85,88].

Collectively, these findings indicate that gemini surfactants provide a versatile platform for advanced fuel and lubricant design, offering simultaneous improvements in stability, performance, and operational reliability that surpass those achievable with traditional single-head surfactants.

## 3. Conclusions

Cationic gemini surfactants exhibit exceptional potential as multifunctional agents within the petroleum sector, driven by their distinctive molecular topology which imparts physicochemical properties far superior to those of conventional monomeric surfactants. Their ability to achieve ultra-low critical micelle concentrations and form stable, viscoelastic interfacial films allows them to operate effectively under the harsh reservoir conditions of high salinity, temperature, and pressure where traditional agents often fail.

In the domain of extraction, these surfactants are proven to significantly enhance oil recovery by reducing interfacial tension to ultra-low values and altering rock wettability, while also stabilizing drilling fluids through robust foam and emulsion formation. Beyond extraction, they offer critical flow assurance benefits in transportation by acting as drag reducers and wax crystal modifiers. A notable advantage of cationic gemini surfactants is their dual-action mechanism in pipeline protection; they not only form protective adsorption layers to inhibit electrochemical corrosion but also possess potent antimicrobial activity that mitigates microbiologically influenced corrosion.

Despite these technical advantages, large-scale industrial deployment remains constrained by high synthesis costs and concerns regarding environmental toxicity. Future advancements must therefore prioritize the development of “green” gemini surfactants utilizing renewable feedstocks and biodegradable spacers. The integration of computational modeling with experimental synthesis promises to accelerate the design of next-generation surfactants that balance high-performance functionality with economic viability and ecological safety.

## Figures and Tables

**Table 1:** Critical micelle concentrations, maximum surface excess and Gibbs free energy values for 1,5-pentamethylene-bis(*N*-alkyl-*N*,*N*-dimethylammonium bromides) *n*-5-*n* [42].

Surfactant	CMC [mol/L]	Γ_max_ × 10^6^ [mol/m^2^]	ΔG°_mic_ [kJ/mol]
6-5-6	1.6 × 10^−1^	1.38	−2.1
8-5-8	4.9 × 10^−2^	1.42	−7.4
10-5-10	7.7 × 10^−3^	1.30	−11.9
12-5-12	1 × 10^−3^	1.45	−16.8
14-5-14	2 × 10^−4^	1.38	−20.8
16-6-16	9.3 × 10^−6^	1.58	−28.3

**Table 2:** Comparative potential of different surfactant types for Enhanced Oil Recovery.

Surfactant Type	Key Performance Characteristics	EOR Potential/Advantages	Limitations/Considerations
Gemini surfactants	Very high surface activity; extremely low CMC; high stability under elevated temperature and pressure; ability to form complex self-assembled structures (e.g., worm-like micelles)	Excellent IFT reduction; improved sweep efficiency; robust performance under harsh reservoir conditions; tunable molecular structure enables optimization for specific reservoirs; strong synergy with nanoparticles and polymers; considered environmentally safer than many alternatives	Production may be more complex; performance strongly depends on spacer architecture and optimized formulation
Zwitterionic surfactants	Tolerant to salinity and temperature variations; often compatible with diverse reservoir chemistries	Can enhance performance when combined with gemini surfactants or nanoparticles; provide stable interfacial films and improve oil mobilization pathways	May require co-surfactants to reach ultra-low IFT; cost may be higher than conventional surfactants
Nonionic surfactants	Good thermal stability; minimal sensitivity to salinity; can modify interfacial properties without strong electrostatic interactions	Improve wettability alteration and can enhance formulations containing gemini surfactants; useful in environmentally compliant formulations	Limited ability to achieve very low IFT on their own; performance depends strongly on hydrophile–lipophile balance (HLB)
Conventional monomeric surfactants (e.g., anionic, cationic)	Moderate surface activity; higher CMC; performance strongly affected by salinity, divalent cations, and temperature	Widely available; established field experience; effective in mild reservoir conditions	Reduced efficiency in harsh environments; limited self-assembly capabilities; weaker interfacial adsorption compared with gemini surfactants; often require higher concentrations and additional stabilizing agents

**Table 3:** Representative numerical performance ranges for different surfactant classes used in EOR.

Surfactant Type	Typical CMC (mM)	Achievable IFT (Oil/Water, mN·m^−1^)	Thermal Stability (°C)	Incremental Oil Recovery
Gemini surfactants	0.01–0.1	10^−3^–10^−4^ (ultra-low IFT)	Up to 120–150	20–35% additional OOIP in core flooding
Zwitterionic surfactants	0.1–1	10^−2^–10^−3^	100–140	10–25% OOIP
Nonionic surfactants	0.1–1.5	10^−1^–10^−2^	80–120	8–18% OOIP
Conventional anionic surfactants	1–10	10^−2^–10^−1^	60–90	5–15% OOIP
Conventional cationic surfactants	1–5	10^−2^–10^−1^	60–100	3–12% OOIP

## Data Availability

The data presented in this study are available in the article.
